# 2-(3,4-Dichlorophenoxy)triethylamine (DCPTA) Sustains Root Activity Through the Enhancement of Ascorbate-Glutathione in Spring Maize (*Zea mays* L.) Under Post-Tasseling Waterlogging

**DOI:** 10.3390/ijms26083698

**Published:** 2025-04-14

**Authors:** Tenglong Xie, Linlin Mei, Xiao-Ge Yang, Meiyu Wang, Qian Zhang, Wei Li, He Zhang, Meng Zhang, Deguang Yang, Jingjie Dou, Xuechen Yang

**Affiliations:** 1 College of Agriculture, College of Animal Science and Technology, Northeast Agricultural University, Harbin 150030, China; tenglongxie@126.com (T.X.); z11425@neau.edu.cn (L.M.); xiaogeyang04@163.com (X.-G.Y.); meiyuwang420@163.com (M.W.); zhq2016@neau.edu.cn (Q.Z.); weili@neau.edu.cn (W.L.); zhanghe@neau.edu.cn (H.Z.); mengzhangad@163.com (M.Z.); deguangyang1967@126.com (D.Y.); 2 State Key Laboratory of Desert and Oasis Ecology, Key Laboratory of Ecological Safety and Sustainable Development in Arid Lands, Xinjiang Institute of Ecology and Geography, Chinese Academy of Sciences, Urumqi 830011, China; doujingjie@nenu.edu.cn

**Keywords:** maize, waterlogging, DCPTA, leaf photosynthesis, root antioxidant system

## Abstract

In Northeast China, waterlogging has emerged as a significant challenge due to climate change, particularly during the June–August period when spring maize (*Zea mays* L.), at the post-tasseling phase, impedes a comprehensive understanding of responses and the development of resistance technologies. 2-(3,4-dichlorophenoxy) triethylamine (DCPTA) is suitable for the entire lifecycle of various economic and food crops, improving crop quality and enhancing stress resistance. The study investigated the ear leaf photosynthesis in relation to the root antioxidant systems’ differential responses of spring maize to waterlogging among the tasseling (VT), vesicle (R2) and dough (R4) stages, and the exogenous DCPTA regulating effect. Results revealed that waterlogging inhibited root physiological activity due to oxidative damage. Consequently, the stomatal restriction and non-stomatal restriction on photosynthesis appeared successively, and R4 was the most sensitive stage. Pretreatment with DCPTA reduced stomatal restriction by maintaining water transfer to the leaf through maintaining root physiological activity via enhanced ascorbate–glutathione cycle. Delayed non-stomatal restriction appeared due to relatively stable chlorophyll content and photosynthetic activities, and VT stage exhibited the highest susceptibility to DCPTA. The study provides a necessary theoretical foundation for comprehending the physiological mechanisms underlying yield formation of spring maize under waterlogging stress in Northeast China, and offers valuable insights for the development of chemical regulation technology.

## 1. Introduction

Maize (*Zea mays* L.), a crucial food resource for a significant proportion of the global population, exhibits pronounced vulnerability to waterlogging stress [1]. Due to its dual utilization as a staple food and animal feed, coupled with its substantial economic importance, maize cultivation in China encompasses an extensive planting area, making it the second-largest global producer of maize. Northeast China, internationally recognized as one of the three major black soil regions, plays a pivotal role in maize production by contributing over 40% of the nation’s total maize yield. However, the availability of water resources in this region is constrained, and exhibits spatial and temporal variations. Attributed to global climate change, there has been a gradual increase in both the frequency and intensity of rainfall events in recent years. Consequently, waterlogging occurs more frequently during June–August when spring maize is at the post-tasseling phase in rain-fed agricultural systems, posing a threat to production stability. For instance, severe waterlogging was observed during extreme rainfall events such as typhoon “Lekima” in 2019, “Bavi”, “Maysak” and “Haishen” in 2020, and “Doksuri” and “Khanun” in 2023. Furthermore, climate models predict an escalation in future rainfall-induced waterlogging events that specifically endanger maize production within Northeast China [2].

Waterlogging severely impacts field crops by depriving roots of oxygen, and disrupting aerobic respiration and energy production [3]. This oxygen deficit forces roots to switch to inefficient anaerobic respiration, generating toxic compounds like ethanol and lactic acid that damage tissues [4]. Prolonged saturation impairs root function, reducing nutrient uptake and causing chlorosis, wilting, and stunted growth [5]. There has been an increasing emphasis on investigating the crop response to waterlogging in recent decades, encompassing peanut [6], sorghum [7] and soybean [8]. Regarding maize, numerous studies have elucidated the molecular-level response mechanism to waterlogging [9,10]. In order to provide theoretical support for practical production, numerous studies on the response of maize to waterlogging have primarily focused on specific ecological regions at the physiological level, particularly those observed in southern China [11]. However, existing research efforts have predominantly focused on summer maize [12] and waxy maize [13], which lack direct reference significance for spring maize production in Northeast China. Furthermore, previous studies on spring maize primarily concentrated on elucidating the mechanism of waterlogging response during the vegetative growth stage, while limited information is available regarding the post-tasseling phase that frequently encounters waterlogging in this region [14]. Therefore, comprehending the physiological mechanism underlying waterlogging stress in spring maize during the post-tasseling phase has become an urgent imperative.

The application of chemical regulation technology has been widely recognized as a highly effective approach for mitigating waterlogging stress. Numerous physiologically active substances, such as 6-benzyladenine [15] and spermidine [16], have demonstrated their ability to enhance waterlogging tolerance in maize. 2-(3,4-dichlorophenoxy) triethylamine (DCPTA) is a representative tertiary amine active substance that has been extensively investigated for its regulatory role in photosynthesis under non-stress conditions. It has been reported to augment chloroplast volume and enhance the activity of ribulose 1,5-bisphosphate carboxylase/oxygenase (RuBPCase) [17]. Additionally, it expedites CO_2_ fixation [18] and is currently widely used in field production. Recent studies have shown that DCPTA can improve maize yield and leaf area [19], and enhance the carbon metabolism pathway [20]. Our previous experiments have demonstrated that exogenous DCPTA promotes maize seedling growth by bolstering leaf photosynthetic capacity [21] and antioxidant levels [22]. Furthermore, transcriptome sequencing revealed significant upregulation of genes associated with nitrogen metabolism in leaves following DCPTA pretreatment [23]. Extensive research projects have demonstrated the beneficial effects of DCPTA treatments on crops exposed to various abiotic stresses, including salinity [24], drought [25], and cold [26] stress. DCPTA, as a novel plant growth regulator, has broad application prospects and significant economic benefits in agricultural production [19,23].

The objective of the present study was to determine the critical stage of waterlogging sensitivity in spring maize during the post-tasseling phase and investigate the similarities and differences in leaf photosynthesis and root antioxidant system response among three developmental stages: tasseling (VT), vesicle (R2), and dough (R4). It was anticipated that (1) waterlogging can cause damage to maize roots, accelerate root aging, and reduce physiological activity, (2) incorporating DCTPA into corn planting could increase maize yield and quality, and enhance crop stress resistance, and (3) the application of DCPTA might exhibit more pronounced effects on waterlogged plants during the R2 stage compared to those at the R4 or VT stages.

## 2. Results

### 2.1. Yield and Agronomic Characters

Waterlogging at various stages during the post-tasseling phase exerted distinct influences on yield components, ultimately leading to variations in overall yield (Table 1). Specifically, the ear and kernel numbers were most significantly impacted at the VT stage, while the kernel weight was most affected at the R4 stage (*p* < 0.05). Consequently, waterlogging at the R4 stage resulted in lower yields compared to the VT and R2 stages.

DCPTA pretreatment enhanced yields under non-stress conditions, and partially mitigated yield declines due to waterlogging stress (*p* < 0.05; Table 1). Meanwhile, DCPTA pretreatment significantly enhanced both shoot and root growth, with a particularly pronounced effect on root development (Table S1). It had a more detrimental impact on plants at the R2 stage compared to the R4 or VT stages (*p* < 0.05).

### 2.2. Water Transport Capacity to Overground Part

**Figure 1:**
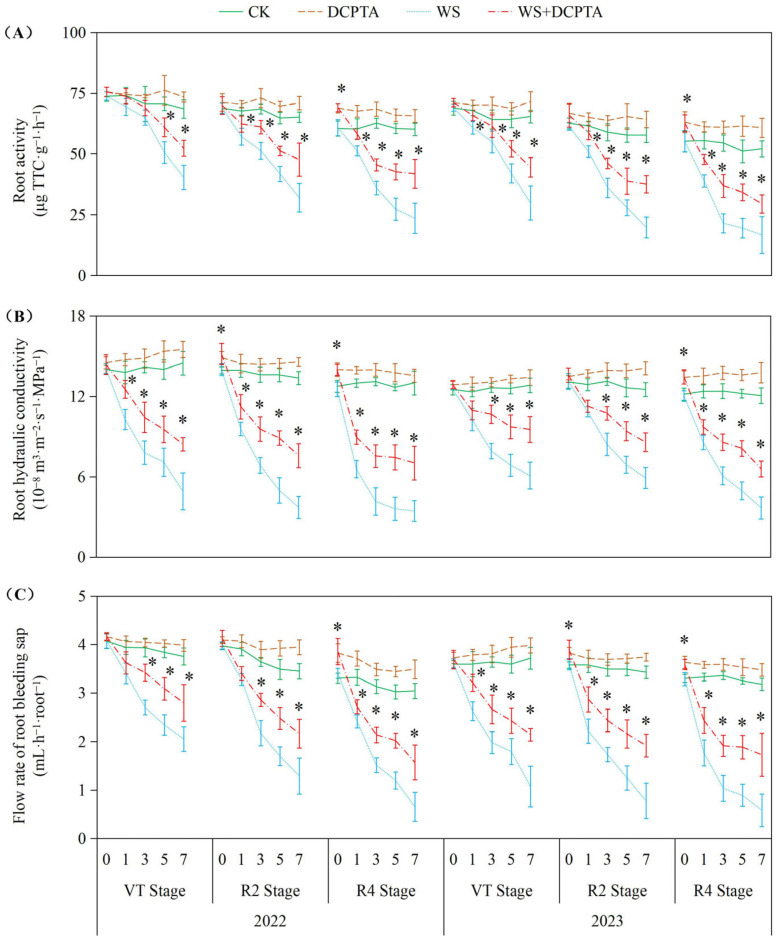
Responses of root activity (**A**), root hydraulic conductivity (**B**) and flow rate of bleeding sap (**C**) to post-tasseling waterlogging and exogenous DCPTA regulating effect. The data represent the means of independent measurements with five replicates, and the standard deviations are indicated by the vertical error bars. “*” on the bars indicates a significant difference at *p* < 0.05 (LSD test) between the waterlogging and waterlogging with DCPTA at each time point.

The data indicate that waterlogging stress significantly impaired root functionality across all measured parameters (root activity, hydraulic conductivity, and bleeding sap flow rate), with the severity of inhibition escalating progressively from the VT to R4 growth stages in both years. Notably, the detrimental effects of waterlogging were more pronounced in 2023 than in 2022, particularly at later stages (R4), where root activity declined by up to 68.02% (vs. 60.83% in 2022) and bleeding sap flow decreased by 81.72% (vs. 78.47% in 2022). DCPTA pretreatment demonstrated substantial mitigation of waterlogging damage, and its protective efficacy diminished at later growth stages, particularly for hydraulic conductivity (45.97% residual inhibition at R4 vs. 41.80% at VT in 2022). Intriguingly, DCPTA exhibited dual functionality—not only alleviating stress but also enhancing root performance under non-stress conditions, with more pronounced promotive effects in 2023 (up to 20.25% increase in root activity vs. 12.19% in 2022) (Figure 1).

### 2.3. Leaf Photosynthetic Performance

**Figure 2:**
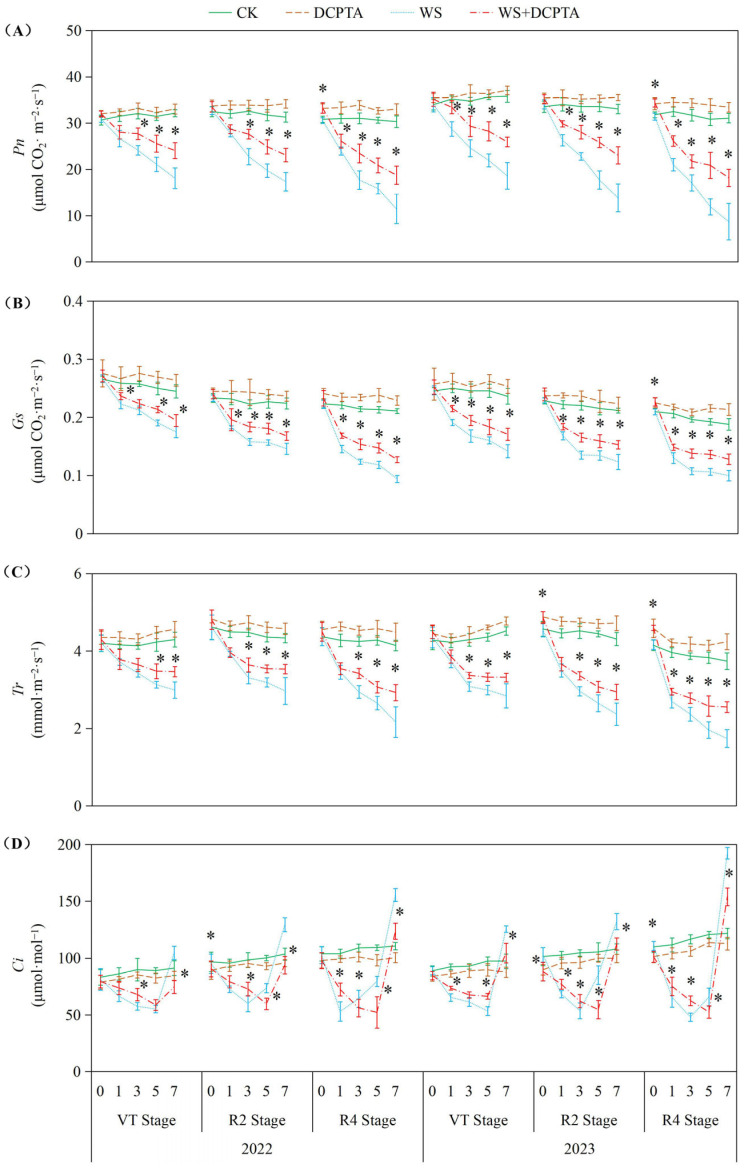
Responses of leaf *Pn* (**A**), *Gs* (**B**), *Tr* (**C**) and *Ci* (**D**) to post-tasseling waterlogging and exogenous DCPTA regulating effect. The data represent the means of independent measurements with five replicates, and the standard deviations are indicated by the vertical error bars. “*” on the bars indicates significant difference at *p* < 0.05 (LSD test) between the waterlogging and waterlogging with DCPTA at each time point.

As depicted in Figure 2, waterlogging stress significantly suppressed photosynthetic parameters (*Pn*, *Gs*, *Tr*) across all growth stages (VT, R2, R4) in both years, with inhibition severity intensifying progressively from VT to R4. For instance, in 2023, *Pn* decreased by up to 71.90% at R4 under waterlogging, compared to 48.04% at VT, highlighting stronger sensitivity during reproductive phases. DCPTA pretreatment effectively mitigated these declines, reducing *Pn* inhibition at R4 from 62.15% (2022) to 38.14% and from 71.90% (2023) to 41.38%, demonstrating its stage-dependent efficacy (R4 > R2 > VT). Notably, DCPTA not only alleviated stress-induced suppression but also actively enhanced photosynthetic performance in non-stressed plants, particularly at R4, where *Pn*, *Gs*, and *Tr* increased by 6.22–9.92%, 5.60–13.65%, and 6.44–13.39% in 2023, respectively. Yearly variations were evident: waterlogging impacts were more pronounced in 2023 (e.g., *Tr* declined by 53.49% vs. 47.79% in 2022 at R4), yet DCPTA’s mitigation effects were also amplified, suggesting heightened utility under severe stress. Under non-stress conditions, *Ci* gradually increased throughout the post-tasseling phase. In contrast, under waterlogging stress, *Ci* initially decreased and then increased. For waterlogging events at the VT, R2 and R4 stages, the minimum *Ci* values occurred on the 5th, 3rd and 1st days in 2022 (5th, 3rd and 3rd days in 2023), respectively.

**Figure 3:**
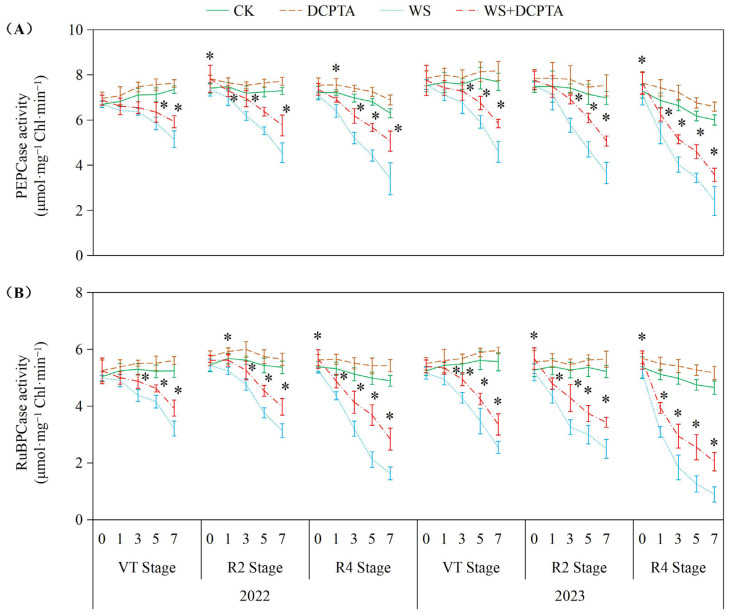
Responses of activities of PEPCase (**A**) and RuBPCase (**B**) to post-tasseling waterlogging and exogenous DCPTA regulating effect. The data represent the means of independent measurements with five replicates, and the standard deviations are indicated by the vertical error bars. “*” on the bars indicates significant difference at *p* < 0.05 (LSD test) between the waterlogging and waterlogging with DCPTA at each time point.

As depicted in Figure 3, waterlogging stress significantly suppressed PEPCase and RuBPCase activities across all tested growth stages (VT, R2, R4) in both years, with inhibition intensifying progressively from VT to R4. RuBPCase exhibited greater sensitivity. DCPTA pretreatment substantially alleviated waterlogging-induced enzyme inhibition. For PEPCase in 2023, declines were reduced to 0.06–27.27% at the R2 stage, and 8.91–40.48% at the R4 stage, compared to 4.56–47.50% and 21.06–59.67% in non-pretreated plants, respectively. Similarly, RuBPCase activity loss was mitigated, e.g., from 80.81% (R4, 2023) to 56.11%. Notably, DCPTA not only counteracted stress effects but also enhanced baseline enzyme activities under normal conditions, elevating PEPCase by 2.33–11.12% and RuBPCase by 2.60–11.12% across both years. As shown in Figures S1 and S2, waterlogging treatment significantly reduced chlorophyll content, *Fv*/*Fm*, *ETR* and *ΦPSII*, while increasing *NPQ* compared to the control group. For DCPTA pretreatment under waterlogging conditions, similar trends were observed with slightly lower percentages.

### 2.4. Root Antioxidant System

**Figure 4:**
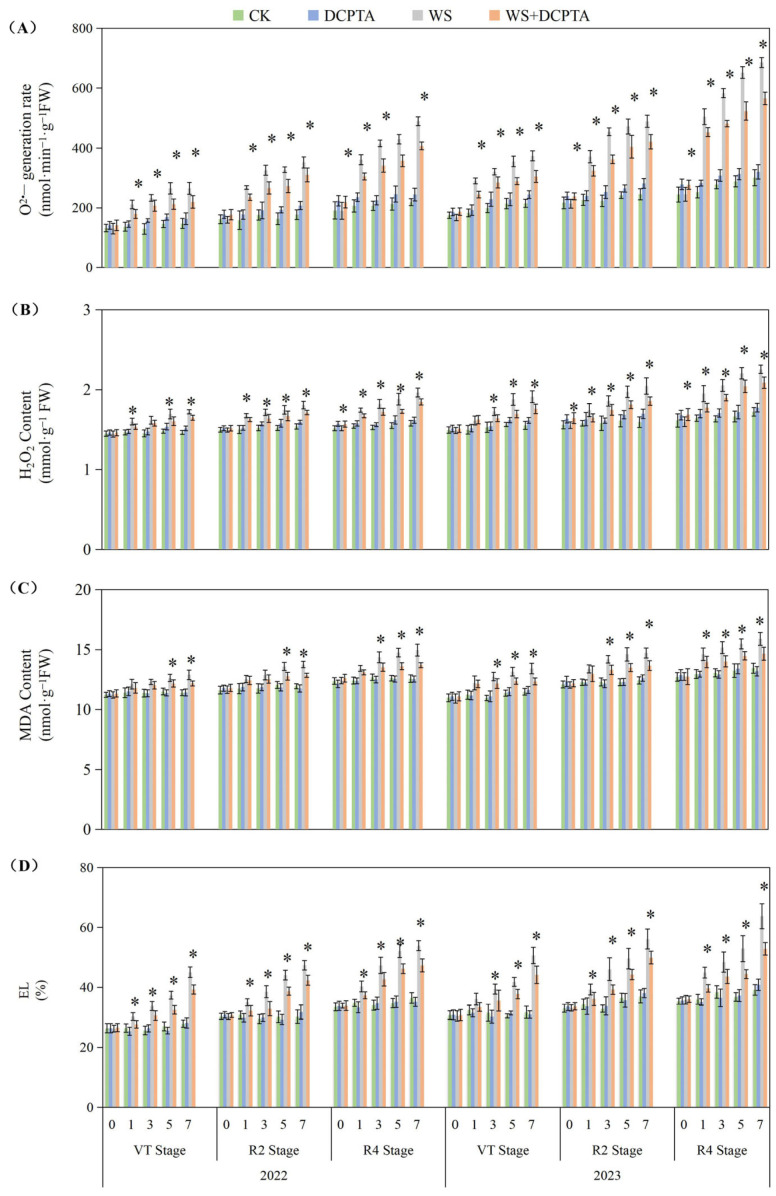
Responses of O^2−^ generation rate (**A**), H_2_O_2_ content (**B**), MDA content (**C**) and EL (**D**) to post-tasseling waterlogging and exogenous DCPTA regulating effect. The data represent the means of independent measurements with five replicates, and the standard deviations are indicated by the vertical error bars. “*” on the bars indicates significant difference at *p* < 0.05 (LSD test) between the waterlogging and waterlogging with DCPTA at each time point.

Waterlogging stress significantly aggravated oxidative damage in plants, manifested through phased increases in O^2−^ generation rate, contents of H_2_O_2_ content, MDA, and EL, with the most severe damage occurring at the R4 stage (Figure 4). After waterlogging treatment in 2022–2023, the O^2−^ generation rate at the R4 stage increased by 74.95–127.79%, while EL peaked at a 62.88% elevation. DCPTA pretreatment effectively mitigated stress-induced damage. However, its protective effects weakened with advancing stages (e.g., the O^2−^ generation rate still increased by 87.86% at R4 in 2023). Notably, under non-stress conditions, DCPTA mildly induced ROS generation (O^2−^ generation rate increased 5.03–21.83%, H_2_O_2_ < 6.54%) without triggering membrane system damage (no significant MDA or EL changes).

**Figure 5:**
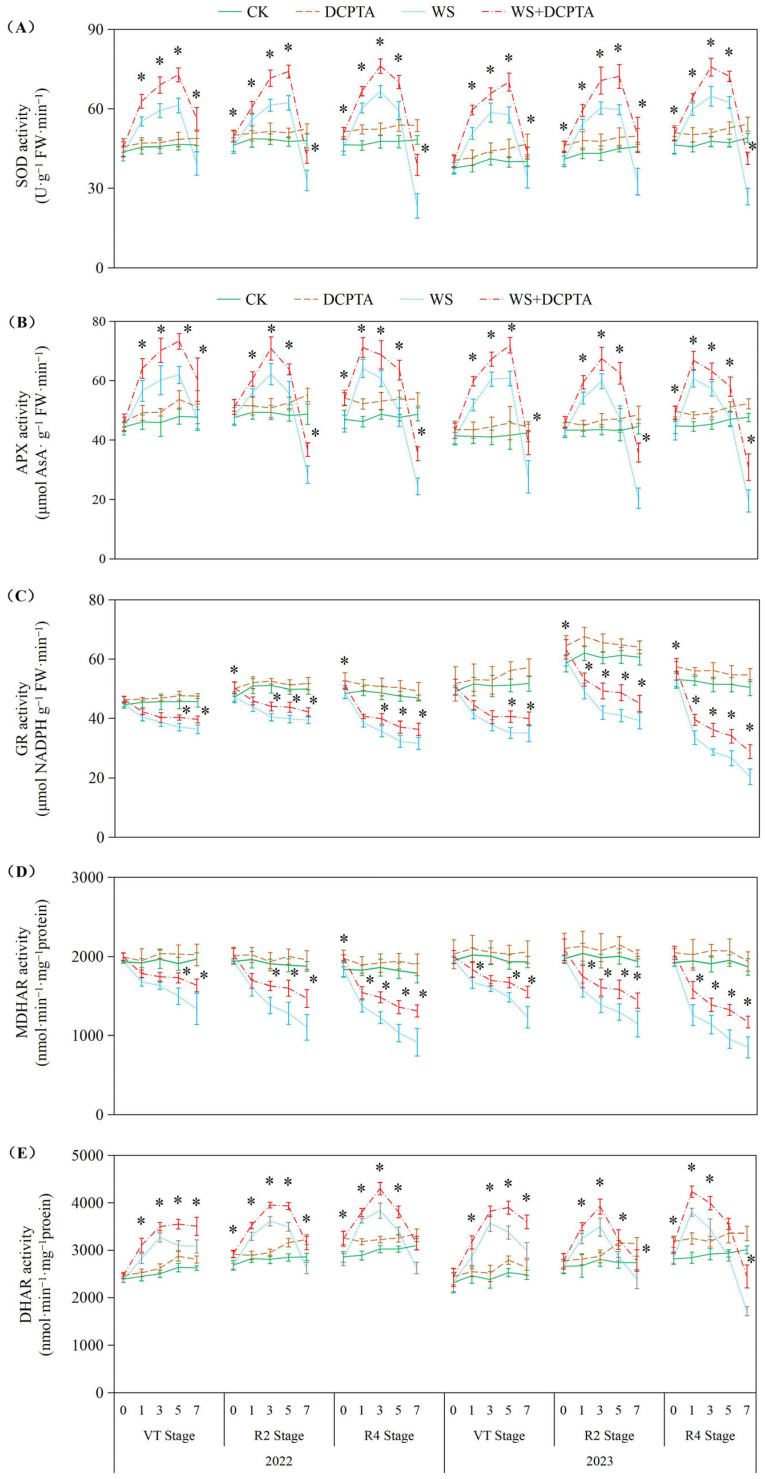
Responses of activities of SOD (**A**), APX (**B**), GR (**C**), MDHAR (**D**) and DHAR (**E**) to post-tasseling waterlogging and exogenous DCPTA regulating effect. The data represent the means of independent measurements with five replicates, and the standard deviations are indicated by the vertical error bars. “*” on the bars indicates significant difference at *p* < 0.05 (LSD test) between the waterlogging and waterlogging with DCPTA at each time point.

In response to waterlogging duration in 2022, the activities of superoxide dismutase (SOD), ascorbate peroxidase (APX) and dehydroascorbate reductase (DHAR) exhibited initial increases followed by decreases; the activities of glutathione reductase (GR), mono dehydroascorbate reductase (MDHAR), peroxidase (POD) and catalase (CAT) exhibited a declining trend, with the decreases becoming more significant as the growth period progressed (*p* < 0.05; Figure 5). GR activity decreased by 11.16–32.44% in 2022 (with inhibition intensifying at later stages) and 19.82–59.49% in 2023. Similarly, MDHAR activity declined by 12.61–48.67% in 2022 and 17.28–54.43% in 2023. Under waterlogging stress, DCPTA pretreatment further enhanced SOD, APX and DHAR activities, and markedly alleviated waterlogging-induced inhibition of GR, MDHAR, POD and CAT. For GR, the reduction narrowed to 6.98–22.43% (2022) and 13.63–42.65% (2023), while MDHAR declines improved to 7.05–26.65% (2022) and 9.24–37.35% (2023). DCPTA alone elevated the enzyme activities to different extents relative to CK.

### 2.5. Ascorbate/Dehydroascorbate (ASA/DHA) and Reduced Glutathione/Oxidized Glutathione (GSH/GSSG) Levels

**Figure 6:**
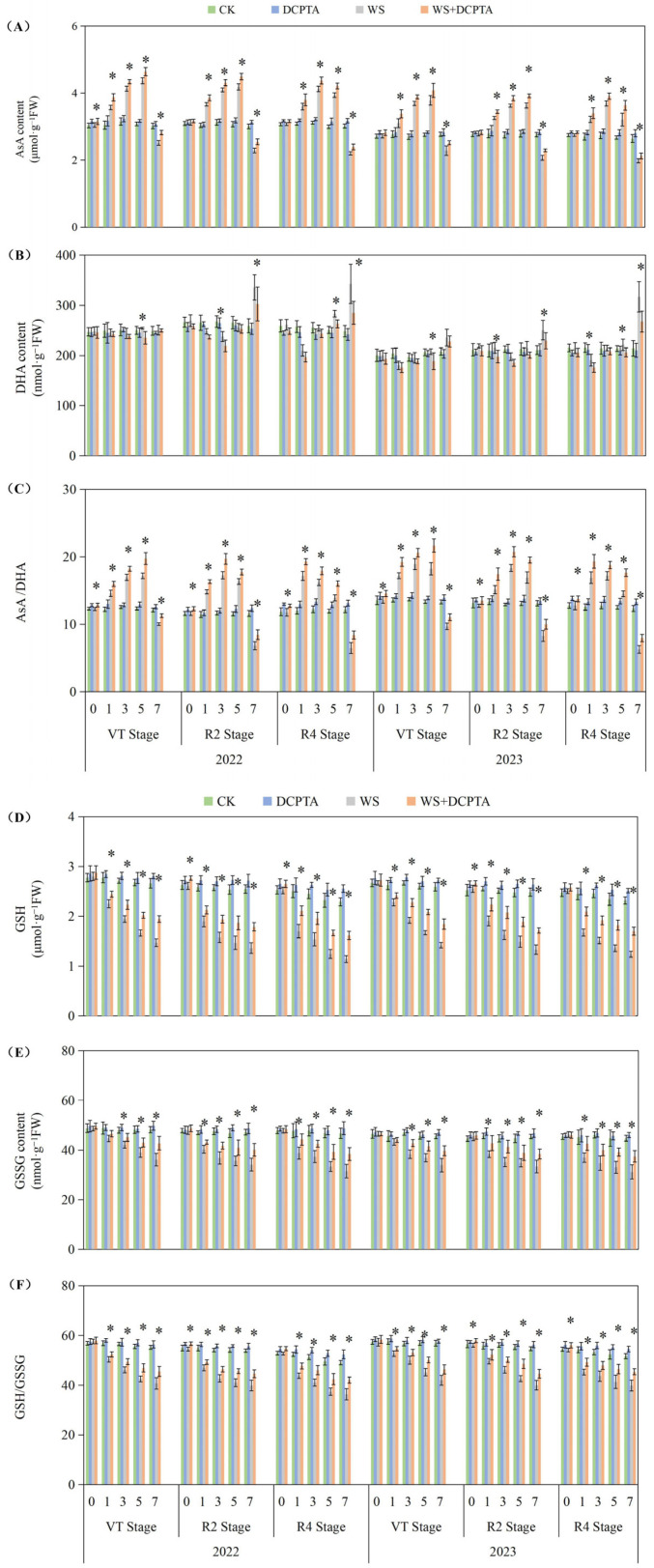
Responses of content of AsA (**A**) and DHA (**B**), AsA/DHA (**C**), GSH (**D**) and GSSG (**E**), and GSH/GSSG (**F**) to post-tasseling waterlogging and exogenous DCPTA regulating effect. The data represent the means of independent measurements with five replicates, and the standard deviations are indicated by the vertical error bars. “*” on the bars indicates significant difference at *p* < 0.05 (LSD test) between the waterlogging and waterlogging with DCPTA at each time point.

The ascorbate/dehydroascorbate (ASA/DHA) ratio exhibited an initial increase followed by a decrease accompanied by the AsA content initially increasing and subsequently decreasing, while the DHA content first decreased and then increased with prolonged waterlogging (Figure 6A–C). The levels of reduced glutathione (GSH) and oxidized glutathione (GSSG) exhibited a declining pattern as waterlogging progressed, with GSSG experiencing a greater decrease, consequently resulting in a continuous downward trend of the GSH/GSSG ratio (Figure 6D–F).

The DCPTA treatment significantly enhanced the magnitude of the increase in AsA content and attenuated the magnitude of the decrease in DHA content during the early stage of waterlogging, moreover, it mitigated the decline in AsA content and restrained the elevation in DHA content during the late stage of waterlogging. Ultimately, it amplified the increase in the AsA/DHA ratio in the early stage while reducing its decrease in the late stage. For VT, R2 and R4 stages, the largest decreases on the 7th day down from 17.25%, 41.16% and 46.70% to 6.92%, 27.15% and 30.77% in 2022; from 27.20%, 36.75% and 49.08% to 16.84%, 23.61% and 35.59%. The DCPTA treatment effectively attenuated the magnitude of the decrease in the contents of GSH and GSSG during the waterlogging stage. Furthermore, it mitigated the decline in the ratio of GSH/GSSG. Under non-stress conditions, treatment with DCPTA resulted in an increase in the levels of both GSH and GSSG, leading to an elevation in the ratio of GSH/GSSG. For VT, R2 and R4 stages, the largest decreases on the 7th day down from 26.17%, 25.94% and 25.81% to 17.57%, 17.31% and 14.21% in 2022; from 26.23%, 26.78% and 22.79% to 18.59%, 18.41% and 12.18% in 2023.

## 3. Discussion

### 3.1. Effects of Post-Tasseling Waterlogging on the Yield of Spring Maize and Exogenous DCPTA Regulating Effect

Maize yield is determined by the interaction of various yield components, including ear number, kernels per ear, and average kernel weight. The response of maize yield to waterlogging varies depending on the growth stage [27]. However, previous studies have reported inconsistent findings regarding the most sensitive period for waterlogging-induced yield reduction. The occurrence of waterlogging during the vegetative growth phase at the V3, V6 and VT stages has been studied by Huang et al. (2022) who suggested that the V6 stage has the greatest impact on yield [13], while Ren et al. (2016) indicated that it is the V3 stage [28]. Considering the frequent occurrence time of waterlogging, this study specifically focuses on investigating the impact of waterlogging at different stages on yield components during the post-tasseling phase to elucidate underlying reasons for varying degrees of yield decline. Panicle differentiation at VT refers to the formation and growth of the reproductive structure, which is crucial for ensuring an adequate nutrient supply to support proper panicle development and maturation. In this study, waterlogging at the VT stage led to significant reductions in kernel number amounting to 13.10% and 14.78% in 2022 and 2023, respectively (Table 1), suggesting that waterlogging-induced losses in yield can be partly attributed to a reduction in kernels per ear resulting from unsuccessful pollination. However, the reductions in yield resulting from waterlogging at the VT stage were not found to be as significant as those observed. During the reproductive stage, approximately 90% of kernel weight is accumulated in the effective kernel-filling phase, with kernel weight being significantly influenced by the level of photosynthesis. At the R2 stage, approximately 12 days after tasseling, the kernel volume in the central region of the ear was essentially established; at the R4 stage, around 26 days after tasseling, the dry weight of kernels in the central region of the ear approached its maximum capacity. The occurrence of waterlogging in 2022 and 2023 resulted in an 8.81% and 10.29% reduction in 1000-kernel weight at the R2 stage, respectively, as well as a significant reduction of 17.10% and 18.69% at the R4 stage, respectively, indicating a substantial impact on assimilate allocation from leaves to kernels which contributed significantly to yield reduction. Additionally, during the reproductive stage, higher kernel filling intensity led to greater damage caused by waterlogging on yield with a more pronounced decrease observed during the R4 stage (27.81% and 29.11% reductions in 2022 and 2023, respectively), compared to the R2 stage (19.02% and 22.13% reductions in yield for years 2022 and 2023, respectively). The decrease in the number of ears affected by waterlogging can be attributed to root lodging observed in this study.

Existing studies have demonstrated that prolonged waterlogging under natural conditions (e.g., lasting more than 15 consecutive days) induces severe hypoxia in maize roots, leading to irreversible membrane lipid peroxidation and photosynthetic system impairment [1,9]. In contrast, our investigation revealed that short-term waterlogging (7 days) caused significantly lower yield reductions (Table 1) compared to the documented impacts of extended waterlogging (average yield losses of 35–45%) [11], establishing waterlogging duration as the critical determinant of maize tolerance thresholds. This highlights the temporal dependency of waterlogging-induced physiological damage and its subsequent agricultural consequences.

Under non-stress conditions, treatment with DCPTA has been shown to enhance yield in maize [19] and other crops, including sugar beet [17], cotton [18], bean [29], tomato [30], guayule [31] and citrus [32]. In our study, we observed consistent improvements in yield of 5.93% and 7.24% for the years 2022 and 2023, respectively. The DCPTA pretreated plants exhibited enhanced tolerance to waterlogging stress compared to the non-treated plants, as evidenced by partial mitigation of the yield decline induced by waterlogging stress. The pretreatment with DCPTA effectively alleviated the reduction in ear number at the VT, R2 and R4 stages, potentially by enhancing plant waterlogging resistance through promoting root growth maintenance. However, its impact on waterlogging-induced damage to kernel number and weight varied among the three stages. It significantly alleviated the decline in kernel number caused by waterlogging at the VT stage, potentially due to its ability to sustain an adequate supply of photoassimilates during panicle differentiation [33]. Additionally, it increased both the number and weight of kernels by facilitating photosynthate mobilization towards unfertilized florets, ensuring a sufficient supply of current photoassimilates compounds for kernel development and suppressing kernel abortion at the R2 stage [34]. Furthermore, it extended leaf photosynthetic activity during kernel filling, alleviating competition among panicle kernels and promoting dry matter accumulation at both R2 and R4 stages [35]. Additionally, the mitigating effect of DCPTA pretreatment on yield reduction diminishes progressively as waterlogging occurs later during the filling process.

### 3.2. Effects of Post-Tasseling Waterlogging on the Leaf Photosynthesis of Spring Maize and Exogenous DCPTA Regulating Effect

Light-harvesting complexes (LHCs) are the predominant protein complexes found on the thylakoid membrane, constituting 50% of their composition as chlorophyll. Consistent with previous research findings, waterlogging stress significantly diminishes light interception by hastening chlorophyll degradation in the ear leaf (Figure S1) [36], and suppresses the maximum photochemical efficiency of PSII, evident from decreased *Fv/Fm* values (Figure S2) [37]. Furthermore, we observed that waterlogging impedes *PSII* electron transport and restricts electron transfers from the reaction center of *PSII* to the primary acceptor plastoquinone (*QA*) and secondary acceptor plastoquinone (*QB*), thereby impeding excitation energy transfer from *LHC* to *PSII* (as evidenced by decreased *ΦPSII* and *ETR*). This limitation in electron transport chains hampers light energy harvesting for ATP generation and reductant production in the form of NADPH during light-dependent reactions. Similar to prior investigations at the V6 stage [38], *Pn* demonstrates a continuous decline under prolonged waterlogging conditions at VT, R2 and R4 stages in this study (Figure 2A). Moreover, we discovered that plants exposed to waterlogging at the R4 stage experienced the most substantial decrease followed by the R2 and VT stages. These results indicate an increasing sensitivity of leaf photosynthesis to waterlogging as growth progresses after tasseling. However, this order differs from the impact of waterlogging damage on yield where R4 > VT > R2, suggesting that maize tassels are more vulnerable to waterlogging than early kernel formation stages.

The impact of waterlogging on *Pn* depends on the severity, magnitude and duration [36], and whether stomatal or non-stomatal factors are the primary cause determined by variations in *Gs* and *Ci* [39]. Consistent with previous studies, waterlogging induces stomatal closure, resulting in a rapid reduction in *Gs* (Figure 2B) [14]. This response can be attributed to the decreased dry weight (Table S1) and impaired metabolic activities of roots (Figure 1A), which may be caused by oxidative damage induced by hypoxic conditions belowground (Figure 4). Consequently, there is limited uptake and transport of H_2_O from roots to ear leaves (Figure 1B,C), directly impacting *Tr* (Figure 2C). During the initial stage of waterlogging, a decrease in CO_2_ uptake is observed while CO_2_ assimilation remains through relatively stable PEPCase and RuBPCase enzymes (Figure 3), resulting in a decline in *Gs* along with a parallel decrease in *Ci*. The primary cause for the decreased *Pn* during this stage is attributed to stomatal factors. As waterlogging progresses, the reduction in *Gs* accompanied by an increase in *Ci*, which may be attributed to reduced CO_2_ assimilation, suggests that non-stomatal restriction predominantly contributes to the decline in *Pn*. Maize is classified as a typical C4 plant belonging to the NADP-malic enzyme subtype. Atmospheric CO_2_ is primarily fixed into oxaloacetate through carboxylation of phosphoenolpyruvate (PEP) via PEPCase in mesophyll cells, and subsequently transported to mesophyll cell chloroplasts. Numerous studies have demonstrated a reduction in PEPCase enzyme activity under waterlogging stress. In this study, the reduction in *Gs* constrained the influx of atmospheric CO_2_ into mesophyll cells, while the diminished activity of PEPCase hindered the fixation of CO_2_ into oxaloacetate through phosphoenolpyruvate carboxylation. The majority of oxaloacetate is subsequently reduced to malate, which is then transported to the bundle sheath cell chloroplasts for decarboxylation, providing both CO_2_ and reducing power. The decreased activity of RuBPCase inhibited the re-fixation of released CO_2_ in the Calvin–Benson cycle. Moreover, we observed both stomatal and non-stomatal responses in relation to waterlogging at different stages of plant development after tasseling. The *Ci* minimum value appeared on the 5th, 3rd, and 1st day in 2022 (on the 5th, 3rd, and 3rd day in 2023) at VT, R2 and R4 stages. These results suggest that non-stomatal limitation occurs earlier at later growth stages under the same waterlogging stress.

Previous studies have demonstrated the efficacy of DCPTA in enhancing plant chlorophyll content [20]. In this study, DCPTA pretreatment was found to augment chlorophyll levels in the ear leaves under non-stressed conditions. However, it partially mitigated the decline in chlorophyll content during waterlogging stress (Figure S1), suggesting its potential involvement in promoting chlorophyll synthesis and improving the effective interception rate of light energy. This enhancement facilitated photon absorption and electron release, resulting in increased *ETR* and conversion of light energy into chemical energy for ATP synthesis (Figure S2). Additionally, DCPTA pretreatment further elevated *NPQ* levels, effectively dissipating excess energy as heat rather than photochemistry. This protective mechanism safeguards photosynthetic machinery against over-excitation and subsequent damage as indicated by increased *NPQ* [40]. The waterlogged plants with DCPTA pretreatment maintain relatively high *Gs* (Figure 2B), ensuring the availability of CO_2_ for the carbon reduction cycle. This may be attributed to sustained root growth (Table S1) and activity (Figure 1A), facilitating abundant supply of H_2_O to leaves, and altering the balance between transpirational loss and root H_2_O uptake. Furthermore, DCPTA pretreatment delays the onset of non-stomatal restriction by preserving the activities of PEPCase and RuBPCase enzymes (Figure 3), facilitating the carboxylation process of phosphoenolpyruvate into oxaloacetate within mesophyll cells, as well as re-assimilating released CO_2_ from malate decarboxylation under waterlogging stress. The maintaining *Pn* could serve as a reasonable strategy for increasing yield (Table 1). Notably, *Pn* of plants exposed to waterlogging at the R4 stage were found to be most susceptible to DCPTA pretreatment.

Prolonged waterlogging may induce programmed cell death in root cortical cells, causing permanent structural damage [28]. In contrast, our study demonstrates that short-term waterlogging primarily transiently limits CO_2_ assimilation through suppression of PEPCase and RuBPCase activities (Figure 3). This distinction suggests that the mitigatory effects of DCPTA observed under short-term waterlogging conditions, particularly its maintenance of enzymatic activities, may not extrapolate to prolonged waterlogging scenarios, thereby necessitating further investigation into its sustained efficacy under extended hypoxic stress.

### 3.3. Effects of Post-Tasseling Waterlogging on the Root Antioxidant System of Spring Maize and Exogenous DCPTA Regulating Effect

The roots, which play a crucial role in facilitating water and nutrient absorption from the soil, are the primary organs that experience a reduction in oxygen tension during waterlogging stress. In this study, the impacts of waterlogging delayed occurrence on root dry weight and activity are amplified (Table 1 and Figure 1). The Reactive oxygen species (ROS) function as signaling molecules that regulate metabolic activities in roots; however, excessive levels of O^2−^ and H_2_O_2_ induced by stress can induce cellular damage by oxidizing proteins, lipids, and other macromolecules within cells [41]. This leads to lipid peroxidation indicated by increased MDA content and membrane deterioration expressed by increased EL as observed in this study (Figure 4). The conversion of O^2−^ to H_2_O_2_ and molecular oxygen O_2_ is facilitated by SOD, which plays a pivotal role in the antioxidant defense system against ROS. Subsequently, H_2_O_2_ is enzymatically converted into H_2_O by antioxidant enzymes such as POD, catalase CAT, and APX. After tasseling, there is a build-up of O^2−^ and H_2_O_2_ under non-stressful conditions, this may be the primary factor contributing to the gradual increase in substrate-induced enzyme activity for SOD and APX, respectively (Figure 5) [42]. Although there was an initial increase followed by a subsequent decrease in SOD activity, plants exposed to waterlogging exhibited a significant elevation in the rate of O^2−^ generation compared to the control group (Figure 4A), indicating that enhanced SOD activity failed to provide adequate protection against O^2−^ accumulation. Contrary to previous studies, in the present study, under waterlogging stress, the activities of peroxidase (POD) and catalase (CAT) exhibited a declining trend, with the decrease becoming more significant as the growth period progressed (Figure 5 and Figure S3), potentially attributed to the downregulation of antioxidant gene expression. This ultimately leads to a reduction in the capacity for scavenging H_2_O_2_. Consequently, the imbalanced ROS production and clearance accelerates root senescence and inhibits root activity (Figure 1A), resulting in reduced capacity for water transportation from roots to leaves as evidenced by decreased root hydraulic conductivity and bleeding sap flow rate (Figure 1B,C), while also disrupting ear leaves photosynthetic performance (Figure 2A). Additionally, in plants exposed to waterlogging, the root activity was observed to be lower at the R4 stage compared to the VT and R2 stages, accompanied by elevated levels of O^2−^ and H_2_O_2_. This suggests that during later growth stages, plants become more susceptible to oxidative stress induced by waterlogging.

In this current investigation, we observed a greater abundance of roots and higher root activity in plants subjected to waterlogging with DCPTA pretreatment compared to those exposed to waterlogging alone (Table 1 and Figure 1). Furthermore, the influence of DCPTA pretreatment on root system performance under waterlogging stress was amplified as the post-tasseling process progressed. Notably, SOD activity was further augmented with DCPTA pretreatment during waterlogging conditions (Figure 5A), facilitating the conversion of O^2−^ into H_2_O_2_. Additionally, DCPTA pretreatment mitigated the decline in enzyme activities of POD and CAT induced by waterlogging (Figure S3), thereby preserving H_2_O_2_ conversion into H_2_O. The AsA–GSH cycle plays a crucial role in maintaining appropriate levels of ROS and cellular redox balance in maize roots [43]. APX primarily functions as the catalyst for converting H_2_O_2_ to H_2_O by utilizing AsA as the reducing agent, both in the chloroplasts and cytosol. This process simultaneously generates two molecules of MDHA, which subsequently disproportionated into DHA [44,45]. In rice seedlings, submerged conditions did not significantly alter total AsA levels compared to air-grown controls [46]. Conversely, under hypoxic conditions, wheat seedling roots exhibited significant increases in reduced forms of AsA [47]. In our study, APX activity initially increased followed by a subsequent decrease during waterlogging stress (Figure 5B). The initial enhancement of APX activity during waterlogging may be attributed to elevated AsA content and the ratio of AsA/DHA levels as part of plants’ adaptive response to increased H_2_O_2_ accumulation (Figure 6).

In the present study, the DCPTA treatment significantly enhanced the magnitude of the increase in AsA content and attenuated the magnitude of the decrease in DHA content during the early stage of waterlogging, moreover, it mitigated the decline in AsA content and restrained the elevation in DHA content during the late stage of waterlogging. Ultimately, it amplified the increase in the AsA/DHA ratio in the early stage while reducing its decrease in the late stage. Under non-stress conditions, pretreatment with DCPTA resulted in an increase in the AsA content, while it had no significant effect on the DHA content. Consequently, there was an elevation observed in the ratio of AsA to DHA. MDHA can be directly reduced to AsA through the catalytic action of MDHAR. DHA is converted to AsA by DHAR, utilizing GSH as a reducing substrate, resulting in the production of GSSG. In this study, MDHAR activity exhibited a continuous decline, while DHAR activity initially increased and subsequently decreased (Figure 5D,E). Several physiological analyses suggested that GSH plays a crucial role in detoxifying ROS in plant roots during hypoxia/anoxia and reoxygenation conditions [48]. The suppressive effect of GR led to a decrease in the GSH/GSSG ratio by inhibiting the reduction in GSSG to GSH, which is associated with cellular GSH levels [49]. Furthermore, DCPTA pretreatment upregulated APX activity and maintained efficient H_2_O_2_ quenching using AsA as an electron donor while oxidizing it into MDHA. Some of the generated MDHA could be reduced to AsA by MDHAR; meanwhile, part of it was converted into DHA. However, DHA can also be reduced to ASA with the involvement of DHAR and GSH. Significant increases in AsA content enhanced plant tolerance against hypoxic stress [50]. In this study, the change in APX activity may be attributed to an increase in AsA content and the AsA/DHA ratio (Figure 6A,C), primarily due to enhanced DHAR activity (Figure 5E) and the GSH/GSSG level (Figure 6F). The significant increase in GSH content improved plant tolerance to hypoxic stress [51]. Furthermore, DCPTA mitigated the downregulation of GR activity induced by waterlogging stress, facilitating efficient regeneration of GSH from GSSG, and thereby promoting stable MDHAR and DHAR activities that facilitated AsA regeneration from DHA. Based on these findings, it can be predicted that the application of DCPTA more effectively scavenges ROS induced by waterlogging stress to maintain normal cellular redox status through stable GR activity. The upregulated expression of GR after DCPTA application increased GSH levels and the GSH/GSSG ratio, consequently leading to a reduction in H_2_O_2_-induced oxidative stress (Figure 4). Therefore, pretreatment with DCPTA alleviated root oxidative stress by regulating enzyme activities involved in the ASA–GSH cycle for excess ROS scavenging.

Although DCPTA alleviated oxidative damage under short-term waterlogging in this study by enhancing activities of antioxidant enzymes (Figure 5) and levels of antioxidant-active substances (Figure 6), prolonged waterlogging may ultimately lead to systemic collapse of ROS scavenging mechanisms, as evidenced by the sustained decline in CAT activity (Figure S3). This progressive dysfunction suggests that DCPTA monotherapy may prove insufficient for maintaining redox homeostasis during extended flooding events. Thus, combinatorial application with other ROS-modulating agents or complementary stress mitigators should be investigated to address the complex oxidative imbalance characteristic of chronic hypoxic stress.

## 4. Materials and Methods

### 4.1. Plant Materials and Experimental Design

The experiments were carried out during the years 2022 and 2023 in Harbin (127°14′ E, 45°78′ N), located in Heilongjiang province, China. This region exhibits a temperate continental monsoon climate. Maize cultivars “ZhengDan 958” and “DCPTA” seeds were procured from Henan Academy of Agricultural Sciences and China Zhengzhou Zhengshi Chemical Limited Company correspondingly.

The experiment was conducted using a randomized complete block design with a split-plot arrangement and five replications. Pits in the field were utilized as experimental containers, measuring 10 m (inner length), 7 m (width), and 1.2 m (height). To maintain consistent soil moisture conditions, plastic sheets covered the inner sides of the pits, while a rain-proof shed ensured that crops relied solely on soil moisture and irrigation throughout the experiment. Prior to planting, soil chemical analysis was performed following Cottenie et al.’s method (1982) [52], and the results are presented in Table 2. Each pit consisted of ten rows, with plant-to-plant distances set at 20 cm and row-to-row distances at 65 cm. Fertilization was carried out before planting by adding ammonium nitrate (33.5% N) at a rate of 8.0 kg per pit, calcium superphosphate (15.5% P_2_O_5_) at a rate of 8.0 kg per pit, and potassium sulfate (48% K_2_O) at a rate of 20 kg per pit. No additional fertilizer was applied after planting.

The seeds were manually sown on 2 May 2022 and 4 May 2023, and harvested on 7 October 2022 and 3 October 2023, respectively. Furthermore, the surrounding ground of the containers was also manually sown with adherence to consistent plant-to-plant and row-to-row distances. Trained personnel implemented measures for plant disease control as well as insect pest management. Throughout both growing seasons (May~October) of 2022 and 2023, a small weather station recorded temperature and precipitation data. In 2022, the average temperature of 14.3 °C in May was slightly lower than normal was the same as the same period of the normal year and 0.2 °C lower than the same period of 2023; 21 °C in June to August was slightly lower, 0.5 °C lower than the same period of normal year and 2023; 10.8 °C in September and October was slightly higher, 0.3 °C higher than the same period of the normal year, 0.2 °C lower than the same period of 2023. In 2022, the average precipitation (65.3 mm) of May was 8.29% more than the same period of the normal year (60.3 mm), close to the same period last year (65 mm); 396.7 mm from June to August was 6.87% more than the same period of the normal year (371.2 mm), and 5.87% less than the same period of 2023 (420 mm); 74 mm in September and October was 16.76% less than that in the same period of the normal year (88.9 mm), and 12.94% more than that in the same period of 2023 (85 mm).

The plants were subjected to the following treatments (Table 3):

(1) Control group (non-waterlogged): Plants received water (10 mL·plant^−1^) at the six-leaf stage, and were not subjected to waterlogging at any growth stage (VT, R2, R4).
(2) DCPTA treatments: Plants were foliar sprayed with 10 mL·plant^−1^ DCPTA solution (35 mg L^−1^, determined based on previous concentration screening) at the six-leaf stage.
(3) Waterlogged treatments: Separate groups of plants were exposed to waterlogging at VT/R2/R4 stages, maintained with flooded water to a level of 4~5 cm above the soil surface for 7 days continuously.

Plant samples were collected at 0, 1, 3, 5 and 7 days after waterlogging treatment for physiological index determination analysis. Yield measurements were conducted at the stage of physiological maturity.

### 4.2. Yeild and Yield Components

For yield determination, an area of 4 m^2^ in each plot was selected for threshing and after threshing, the grain yield was measured.

Yield (Kg·mu^−1^) = ears (m^−2^) × kernels (ear^−1^) × 1000-kernel weight (g) × 667 (m^2^)

### 4.3. Agronomic Characters

At the conclusion of the 7-day waterlogging stress period, plants were randomly selected from each pit and divided into shoot and root components. Subsequently, they were subjected to oven drying at 105 °C for 45 min followed by a holding temperature of 80 °C for 48 h. Dry weights of both roots and shoots were determined immediately thereafter. The average values obtained from ten plants within one pit constituted one replicate.

### 4.4. Root Activity, Root Hydraulic Conductivity and Flow Rate of the Root-Bleeding Sap

The root activity was assessed using the Plant Root (dehydrogenase) Activity Detection kit (HK-BC5270) through 2,3,5-triphenyltetrazolium chloride (TTC) colorimetry in accordance with the manufacturer’s instructions. Root hydraulic conductivity was determined by employing the Scholander pressure chamber method. The plants were cut 10 cm from the ground, a centrifuge tube with cotton was used to cover the incision, and a plastic bag was used to seal it to prevent water loss as well as dust and insects. The cotton samples were collected from 8:00 p.m. to 8:00 a.m., the exudate volume was estimated based on the increase in cotton weight, and the root bleeding intensity (mL h^−1^ root^−1^) was estimated according to the increase in cotton weight [53].

### 4.5. ROS Damage Characters

The formation rate of O^2−^, H_2_O_2_ content, MDA content and EL were determined according to the methods of Elstner and Heupel (1976) [54], Jana and Choudhuri (1982) [55], Heath and Packer (1968) [56] and Lutts et al. (1995) [57], respectively.

### 4.6. Antioxidant Enzyme Activities

To extract the antioxidant enzymes, frozen root samples (0.5 g) were homogenized using a chilled mortar and pestle with 8 mL of ice-cold 50 mM phosphate buffer (pH 7.8) and then immediately centrifuged (12 000× *g* for 20 min at 4 °C). Phosphate buffer was added to the supernatant to a final volume of 5 mL, which was used for the antioxidant enzyme activity assays with a UV-visible spectrophotometer (Shimadzu, Kyoto, Japan).

The activities of SOD (EC 1.15.1.1), POD (EC 1.11.1.7), and CAT (EC 1.11.1.6) were determined using the nitrogen blue tetrazole photochemical reduction method [58], guaiacol method and H_2_O_2_ dissipation method [56], respectively. The activities of APX, (EC 1.11.1.11), GR (EC 1.6.4.2), MDHAR (EC 1.6.5.4) and DHAR (EC 1.8.5.1) were measured by monitoring the decrease in AsA absorbance at 290 nm [59], the decrease in absorbance at 340 nm caused by NADPH oxidation [60], the decrease in absorbance at 340 nm caused by NADH oxidation [61], the increase in absorbance at 265 nm caused by AsA formation [59].

### 4.7. AsA/DHA and GSH/GSSG in Roots

The levels of AsA, DHA, GSH and GSSG in maize roots were quantified using the corresponding kits (Solarbio, Beijing, China) following the methodology described in a previous study [62].

### 4.8. Chlorophyll Content

The sampling for chlorophyll pigments analyses was carried out between 10:00 a.m. and 2:00 p.m. Chlorophyll contents were extracted by grinding samples in 10 mL of 80% acetone and centrifuged at 1000 r min^–1^ at 4 °C for 3 min. Then, these samples were placed in 10 mL of 80% aqueous acetone solution in the darkness at room temperature for 24 h. The supernatant was then separated and analyzed with a spectrophotometer (SpectraMax i3x from Bischofshofen, Austria) at wavelengths of 652 nm to compute the chlorophyll content (mg·g^−1^).

### 4.9. Chlorophyll Fluorescence Parameters and Gas Exchange

The chlorophyll fluorescence parameters were measured with a pulse-amplitude-modulated (PAM-2500) fluorometer (Walz, Nuremberg, Germany). The leaves from each treatment were subjected to dark adaptation for more than 20 min. *Fv*/*Fm*, *ETR*, *ΦPSII* and *NPQ* were calculated as described by Maxwell and Johnson (2000) [63].

### 4.10. Gas Exchange

The *Pn*, *Tr*, *Gs* and *Ci* values were determined with a portable photosynthesis system (LI-6400XT; LI-COR Biosciences, Lincoln, NE, USA) between 13:00~14:00 p.m. The 6 cm^2^ leaf chamber was used, and the photo flux density was 1000 μmol m^−2^ s^−1^.

### 4.11. Carbon Metabolism-Related Enzyme Activity

The ear leaves from each treatment were sampled on the following day and immediately frozen in liquid nitrogen before being held at −80 °C. Subsequently, 0.5 g of fresh samples were homogenized using phosphate acid buffer (pH = 7.4), followed by centrifugation at 4000 r min^−1^ for 20 min to collect the supernatants for analysis. The activities of PEPCase and RuBPcase were determined using ELISA kits according to the manufacturer’s instructions. Each treatment was replicated five times biologically.

### 4.12. Statistical Analysis

All of the data were tested for normality and homogeneity of variance before analysis. The means were compared by ANOVA and least significant differences (LSD) tests at the 5% level. Data used in this study were analyzed using SPSS 18 (IBM Corporation, Armonk, NY, USA). Microsoft Excel 2010 was used to draw the figures.

## 5. Conclusions

Waterlogging can lead to damage to the corn root system, resulting in reduced water and nutrient absorption, weakening the overall health of the plant, resulting in delayed crop maturity or even premature death. Our results demonstrated that waterlogging not only decreased root weight, but also increased accumulation of O^2−^ and H_2_O_2_ due to confused changes of antioxidant enzyme activities and nonenzymic antioxidant contents, initiating lipid peroxidation and membrane deterioration, accelerated root senescence and decreased physiological activity. The stomatal restriction and non-stomatal restriction on photosynthesis appeared successively, and R4 was the most sensitive stage. DCPTA alleviated stomatal restriction by sustaining root activity through ascorbate-glutathione enhancement in Spring Maize under post-tasseling waterlogging. Meanwhile, the chlorophyll content and photosynthetic activity are relatively stable, with delayed non-stomatalnon stomatal limitation, and the VT period has the highest sensitivity to DCPTA. DCPTA has been widely used in agriculture due to its unique physiological functions. It can increase crop yield and quality, and enhance crop stress resistance. Future research should focus on studying the molecular targets and signaling pathways of DCPTA in plant cells, clarifying the interactions between DCPTA and various biomolecules in plants, and conducting in-depth studies on the synergistic effects of DCPTA with fertilizers, pesticides, other plant growth regulators, and other agricultural inputs to improve crop yield and quality, reduce the use of other agricultural inputs, and lower costs and environmental pollution risks.

The conclusions of this study are derived from short-term waterlogging stress (7 days) at specific post-tasseling stages, whereas natural waterlogging events may involve multi-phase sequential stresses or prolonged durations. Although DCPTA exhibited significant mitigation effects under experimental conditions, its field application requires comprehensive evaluation integrating waterlogging duration, soil types, and climatic conditions. Future investigations could simulate extended waterlogging scenarios (e.g., 15–20 days) while monitoring DCPTA’s dynamic responses to validate its broad-spectrum stress resistance.

## Figures and Tables

**Table 1:** Changes in yield components of maize in 2022 and 2023.

Year	Stage	Treatment	Number of Ears (ears·mu^–1^)	Number of Kernels (kernels·ear^–1^)	1000-Kernel Weight (g)	Yield (kg·mu^–1^)
2022	/	CK	4661 ± 84	574 ± 17	316.33 ± 7.41	846.27 ± 22.80
		CK + DCPTA	4691 ± 108	587 ± 10	325.16 ± 4.50	896.41 ± 35.47
	VT	Waterlogging	4309 ± 47	499 ± 15	300.70 ± 3.51	646.50 ± 16.49
		Waterlogging + DCPTA	4436 ± 61 *	545 ± 16 *	312.80 ± 6.01 *	756.53 ± 29.47 *
	R2	Waterlogging	4411 ± 62	539 ± 18	288.47 ± 5.90	685.28 ± 19.81
		Waterlogging + DCPTA	4559 ± 84 *	558 ± 18	307.53 ± 8.29 *	782.11 ± 34.94 *
	R4	Waterlogging	4494 ± 57	518 ± 20	262.24 ± 9.22	610.91 ± 33.23
		Waterlogging + DCPTA	4595 ± 49	533 ± 21	282.42 ± 4.96 *	691.70 ± 39.88 *
2023	/	CK	4554 ± 81	558 ± 17	309.73 ± 7.72	786.01 ± 16.82
		CK + DCPTA	4611 ± 88	572 ± 8	319.22 ± 3.98	842.85 ± 35.29
	VT	Waterlogging	4180 ± 58	475 ± 25	292.74 ± 4.60	581.36 ± 30.76
		Waterlogging + DCPTA	4314 ± 57 *	537 ± 17 *	305.40 ± 6.82 *	707.55 ± 36.48 *
	R2	Waterlogging	4273 ± 50	515 ± 12	277.92 ± 8.28	612.08 ± 23.75
		Waterlogging + DCPTA	4437 ± 104 *	544 ± 18 *	299.73 ± 10.28 *	723.54 ± 46.36 *
	R4	Waterlogging	4425 ± 69	500 ± 17	251.84 ± 8.33	557.25 ± 34.74
		Waterlogging + DCPTA	4473 ± 44	524 ± 19 *	280.62 ± 7.08 *	657.84 ± 31.19 *

The data represent the means of independent measurements with five replicates, values with the “*” indicated significantly different at *p* < 0.05 (LSD test) between the waterlogging and waterlogging with DCPTA in each stage.

**Table 2:** Chemical properties (mg kg^−1^) of the used soil.

Year	pH	HCO_3_^−^ + CO_3_^2−^	Cl^−^	SO_4_^2−^	Ca^2+^	Mg^2+^	Na^+^	K^+^	N	P
2022	7.2	205.3	284.6	472.9	86.9	38.5	4.2	29.7	16.7	3.6
2023	7.1	202.7	302.5	445.7	91.2	41.3	4.1	31.2	14.6	3.9

**Table 3:** Experimental treatments.

Treatment	Foliar Spray (Six-Leaf Stage)	Waterlogging Stage	Waterlogging Duration
Control	Water	None	0 days
DCPTA	DCPTA (35 mg·L^−1^)	None	0 days
Waterlogging + Water	Water	VT, R2, or R4	7 days
Waterlogging + DCPTA	DCPTA (35 mg·L^−1^)	VT, R2, or R4	7 days

## Data Availability

As the data involves student graduation, it is currently not convenient for public download. If needed, please contact the corresponding author for access.

## Supplementary Materials

The following supporting information can be downloaded at: https://www.mdpi.com/article/10.3390/ijms26083698/s1.
