# Supplementary material for: 2-(3,4-Dichlorophenoxy)triethylamine (DCPTA) Sustains Root Activity Through the Enhancement of Ascorbate-Glutathione in Spring Maize (Zea mays L.) Under Post-Tasseling Waterlogging
Source: Int J Mol Sci. 2025 Apr 14;26(8):3698. doi: 10.3390/ijms26083698 (PMC12027506; doi:10.3390/ijms26083698)
Supplement: Supplementary file 1 [file ijms-26-03698-s001.zip › ijms-3537976-supplementary.pdf]

Table S1. Changes in dry matter accumulation of maize in 2022 and 2023

Stage	Treatment	Shoot dry weight(g)		Root dry weight(g)	
		**2022**	**2023**	**2022**	**2023**
VT stage	CK	100.64±2.06	96.47±4.53	6.15±0.33	5.97±0.25
	CK+DCPTA	104.37±4.69	100.58±3.17	6.54±0.24	6.42±0.26d
	VT	85.83±3.74	80.31±4.26	4.89±0.28	4.59±0.25
	VT+DCPTA	92.61±3.48	90.29±3.54*	5.58±0.24*	5.31±0.24*
R2 stage	CK	240.35±7.18	240.82±7.71	9.43±0.50	8.89±0.47
	CK+DCPTA	253.49±5.26	256.87±8.30	10.13±0.54	9.67±0.44
	VT	193.22±9.54	189.21±6.80	6.26±0.89	5.74±0.54
	VT+DCPTA	204.74±7.60*	199.61±7.34*	7.57±0.62*	6.96±0.56*
R4 stage	CK	340.57±10.76	310.05±6.51	9.43±0.51	7.93±0.81
	CK+DCPTA	363.78±6.19	334.56±9.68	10.21±0.67	8.68±0.57
	VT	276.95±10.14	249.68±8.38	6.59±0.62	5.34±0.79
	VT+DCPTA	294.36±8.05*	270.39±9.48*	7.68±0.51*	6.59±0.62*

The data represent the means of independent measurements with five replicates, values with the "*" indicated significantly different at $p < 0.05$ (LSD test) between the waterlogging and waterlogging with DCPTA in each stage.

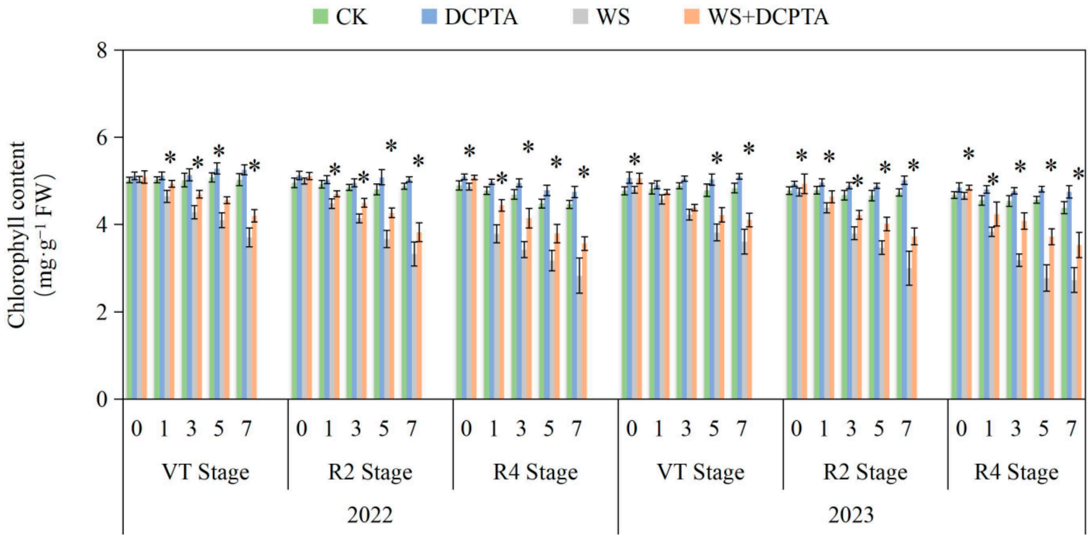

Figure S1. Responses of leaf chlorophyll content to post-tasseling waterlogging and exogenous DCPTA regulating effect. The data represent the means of independent measurements with five replicates, and the standard deviations are indicated by the vertical error bars. “*”on the bars indicates a significant difference at $p < 0.05$ (LSD test) between the waterlogging and waterlogging with DCPTA at each time point.

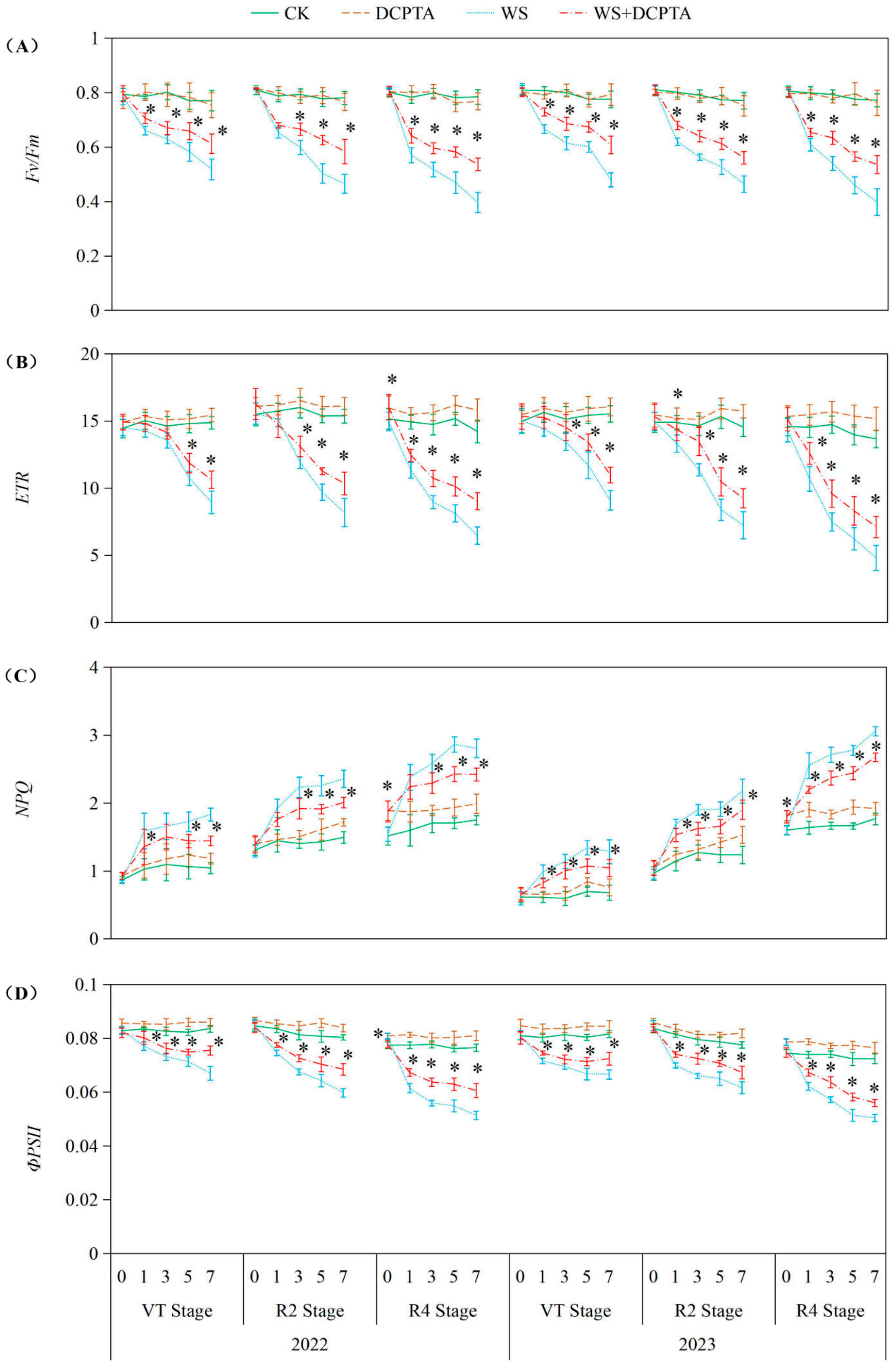

Figure S2. Responses of leaf *Fv*/*Fm* (A), *ETR* (B), *NPQ* (C) and *ΦPSII* (D) to post-tasseling waterlogging and exogenous DCPTA regulating effect. The data represent the means of independent measurements

with five replicates, and the standard deviations are indicated by the vertical error bars. “*”on the bars indicates a significant difference at $p < 0.05$ (LSD test) between the waterlogging and waterlogging with DCPTA at each time point.

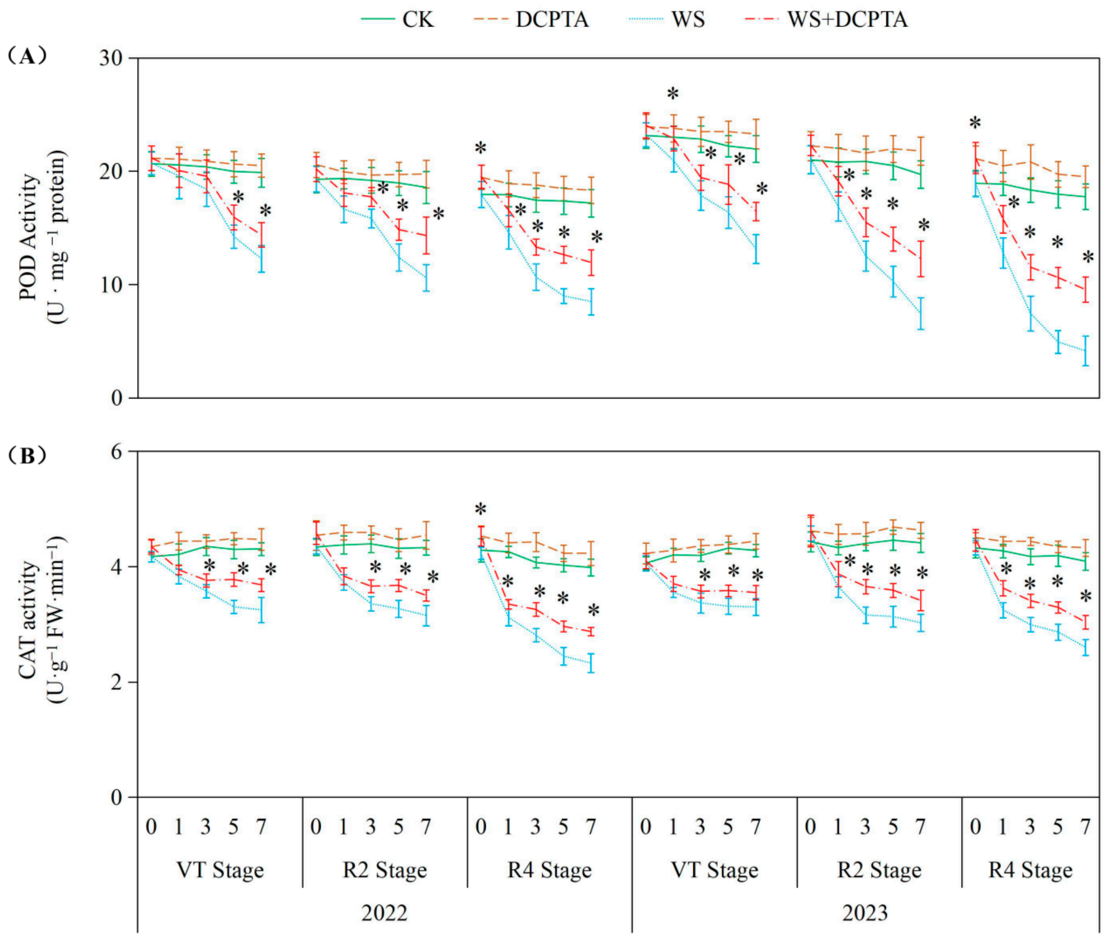

Figure S3. Responses of activities of POD (A) and CAT (B) to post-tasseling waterlogging and exogenous DCPTA regulating effect. The data represent the means of independent measurements with five replicates, and the standard deviations are indicated by the vertical error bars. “*”on the bars indicates a significant difference at $p < 0.05$ (LSD test) between the waterlogging and waterlogging with DCPTA at each time point.
